# Using patient-reported outcome measures and patient-reported experience measures to elevate the quality of healthcare

**DOI:** 10.1093/intqhc/mzad098

**Published:** 2023-12-26

**Authors:** Pedro Casaca, Willemijn Schäfer, Ana Beatriz Nunes, Paulo Sousa

**Affiliations:** NOVA National School of Public Health, Public Health Research Centre, Comprehensive Health Research Center, CHRC, NOVA University Lisbon, Lisbon, Portugal; Nivel, The Netherlands Institute for Health Services Research, Netherlands; NOVA National School of Public Health, Public Health Research Centre, Comprehensive Health Research Center, CHRC, NOVA University Lisbon, Lisbon, Portugal; NOVA National School of Public Health, Public Health Research Centre, Comprehensive Health Research Center, CHRC, NOVA University Lisbon, Lisbon, Portugal

The path to exceptional healthcare quality is understanding patients’ experiences and needs and the subsequent integration of these insights into clinical practice [1]. The importance of the patient voice is also reflected in the 2023 World Patient Safety Day theme: ‘Engaging patients for patient safety’. One way to understand patient experiences and needs is by using patient-reported outcome measures (PROMs) and patient-reported experiences measures (PREMs). PROMs measure patients’ views of their symptoms, their functional status, and their health-related quality of life at a single point in time and are collected through short, self-completed questionnaires. This health status information indicates the outcomes or quality of care [2]. PREMs measure patients’ perception of their personal experience of receiving healthcare. These questionnaire-based instruments ask patients to report on the extent to which certain predefined processes occurred during an episode of care [3].

Globally, healthcare systems have seen multiple efforts to adopt PROMs and PREMs, but their implementation often remains localized rather than systemic. Additionally, a clear gap exists between PROMs and PREMs used in temporary research projects and those continuously utilized for quality improvement or routine care, leading to wasteful practices and missed opportunities [4]. Furthermore, current healthcare metrics, though comprehensive, often miss patient perspectives. Involving patients in regularly reporting their outcomes and experiences process establishes metric relevance and enhances data quality.

Alignment between patients, healthcare professionals, policymakers, and researchers is key to unlocking the full potential of PROMs and PREMs and improving the value of care. There are multiple benefits of incorporating PROMs and PREMs into care routines and as part of quality improvement initiatives, including:

**Implementing person-centred care:** Healthcare professionals gain insights into individual patient outcomes and experiences of their patients by routinely collecting PROMs and PREMs. The data can enable them to conduct more thorough recollections and allow for more person-centred care. Integrating PROMs and PREMs into electronic health records can support the active use of data for person-centred care.

**Driving quality improvement and standardized care**: Aggregated PROMs and PREMs data can be utilized for systematic evaluation, supporting quality enhancement, and benchmarking. These activities contribute to a comprehensive adoption of a value-based healthcare approach. Such mechanisms help drive better care standards within and across institutions.

**Informative framework for research and policymaking**: Beyond immediate clinical care, PROMs and PREMs data are valuable for researchers and policymakers. Through evaluation and analysis knowledge can be produced to develop health practices and policies to better align with patient experiences, needs, and outcomes.

Integrating PROMs and PREMs is essential to elevating care quality and patient involvement. Using the Donabedian framework, which evaluates quality based on structures, processes, and outcomes, PROMs and PREMs emerge as vital components when assessing care delivery and outcomes. PROMs and PREMs can be embedded as part of regular quality improvement and monitoring by care providers and facilities, and in research projects. The recent Horizon Europe Framework Programme (HORIZON) call ‘Ensuring access to innovative, sustainable and high-quality health care’ highly valued projects that prioritized patients’ perspectives. Research projects such as BE-SAFE, SafePolyMed, DELIVER, and SAFEST have championed this approach, embedding PROMs and PREMs into their research methodologies. The International Survey of People Living with Chronic Conditions (PaRIS survey), spearheaded by the Organisation for Economic Co-operation and Development (OECD), aims, among other things, to map existing PROMs and PREMs used for primary care users aged ≥45 years living with chronic conditions on a global scale [5]. The development and implementation of the PaRIS survey involved extensive co-development, incorporating input from policymakers, patients, healthcare providers, and researchers. However, balancing standardization with adaptations to each country’s culture and healthcare system posed a significant challenge. The initiative underscores the essential role of co-development and stakeholder engagement in balancing scientific rigour and practical implementation in international health data collection efforts. It implies that international collaborations like PaRIS can enhance data systems and fast-track the application of patient-reported measures in primary care for quality and safety enhancement [6].

Implementing PROMs and PREMs is demanding and the path to implementation is complex. Their use often incurs extra costs and time, particularly with traditional paper-based methods. There are also concerns about low response rates and potential biases due to lower participation of certain groups, namely hard-to-reach populations. However, it is the experiences of these very populations that is necessary to understand if we are to improve care delivery and outcomes. Rather than avoiding, we must embrace and overcome the challenges, sharing our experiences, strategies, and learnings to make it easier for others. We know the long-term advantages far outweigh the initial challenges. Strategies to consider, where possible, include switching to electronic PROMs and PREMs, allowing patients to use their own devices or providing dedicated devices; any action that can streamline the process should be considered [7]. Similarly, approaches such as digital reminders can boost response rates [8] while customizing PROMs and PREMs ensures inclusivity for all sociodemographic groups, safeguarding equity [9].

Integrating PROMs and PREMs must not be seen as a mere punctual measure but as an imperative to better understand and improve how healthcare is delivered. A combined effort promoting patients’ education and health literacy, inclusion in care routines and a systemic approach based on continuous assessment and improvement is vital to elevate the quality of care. Healthcare quality is achieved when patients and providers collaboratively steer the patient’s journey. Thus, PROMs and PREMs offer the means to ensure patients’ voices are central in healthcare’s intricate symphony: seamless implementation is the future of equitable, safe, patient-centred, high-quality care.
